# Prospective, double-blind, placebo-controlled randomized trial of cimetidine in gastric cancer

**DOI:** 10.1038/sj.bjc.6690457

**Published:** 1999-12

**Authors:** M J S Langman, J A Dunn, J L Whiting, A Burton, M T Hallissey, J W L Fielding, D J Kerr

**Affiliations:** Department of Medicine andClinical Trials Unit, CRC Institute for Cancer Studies, Clinical Research Block, The Medical School, Birmingham, Edgbaston B15 2TA, UK; Department of Surgery, Queen Elizabeth Hospital, Birmingham B15 2TH, UK

**Keywords:** cimetidine, gastric cancer, randomized, clinical trial, H_2_-antagonists

## Abstract

Cimetidine is thought to inhibit suppressor T-lymphocyte function and preliminary evidence from a randomized trial indicated that it might prolong survival for patients with operable and inoperable gastric cancer. The British Stomach Cancer Group conducted a randomized, double-blind, placebo-controlled trial examining the effects of cimetidine (400 mg or 800 mg twice a day) on the survival of patients with early (stages I, II and III: *n* = 229) and advanced (stages IVa and IVb: *n* = 201) gastric cancer. The primary end point was death. A total of 442 patients were randomized by 59 consultants in 39 hospitals between February 1990 and March 1995. Log-rank survival analysis was used to assess differences between the groups. Three hundred and forty patients died during the study: 166 (49%) in the cimetidine treatment groups and 174 (51%) in the placebo groups. Median survival for patients receiving cimetidine was 13 months (95% confidence interval (CI) 9–16 months) and 11 months in the placebo arm (95% CI 9–14 months). There was no significant difference in survival between the two treatment groups (*P* = 0.42) or between different doses of cimetidine tablets (*P* = 0.46). Five-year survival of those patients randomized to cimetidine was 21% compared to 18% for those patients randomized to placebo. Cimetidine at a dose of 400 mg or 800 mg twice a day does not have a significant influence on the survival of patients with gastric cancer compared to placebo. © 1999 Cancer Research Campaign


*British Journal of Cancer* (1999) **81**(8), 1356–1362
© 1999 Cancer Research Campaign
Article no. bjoc.1999.0852

### Prospective, double-blind, placebo-controlled randomized trial of cimetidine in gastric cancer

**MJS Langman[1], JA Dunn[2], JL Whiting[3], A Burton[2], MT Hallissey[3], JWL Fielding[3] and DJ Kerr[2] on behalf of the British Stomach Cancer Group**

[1]Department of Medicine and [2]Clinical Trials Unit, CRC Institute for Cancer Studies, Clinical Research Block, The Medical School, Edgbaston, Birmingham B15 2TA, UK; [3]Department of Surgery, Queen Elizabeth Hospital, Birmingham B15 2TH, UK

**Summary** Cimetidine is thought to inhibit suppressor T-lymphocyte function and preliminary evidence from a randomized trial indicated that it might prolong survival for patients with operable and inoperable gastric cancer. The British Stomach Cancer Group conducted a randomized, double-blind, placebo-controlled trial examining the effects of cimetidine (400 mg or 800 mg twice a day) on the survival of patients with early (stages I, II and III: $n$ = 229) and advanced (stages IVa and IVb: $n$ = 201) gastric cancer. The primary end point was death. A total of 442 patients were randomized by 59 consultants in 39 hospitals between February 1990 and March 1995. Log-rank survival analysis was used to assess differences between the groups. Three hundred and forty patients died during the study: 166 (49%) in the cimetidine treatment groups and 174 (51%) in the placebo groups. Median survival for patients receiving cimetidine was 13 months (95% confidence interval (CI) 9–16 months) and 11 months in the placebo arm (95% CI 9–14 months). There was no significant difference in survival between the two treatment groups ($P$ = 0.42) or between different doses of cimetidine tablets ($P$ = 0.46). Five-year survival of those patients randomized to cimetidine was 21% compared to 18% for those patients randomized to placebo. Cimetidine at a dose of 400 mg or 800 mg twice a day does not have a significant influence on the survival of patients with gastric cancer compared to placebo. © 1999 Cancer Research Campaign

**Keywords:** cimetidine; gastric cancer; randomized; clinical trial; $H_2$-antagonists

Cancer of the stomach remains endemic in many parts of the world and continues to present a difficult therapeutic challenge. Although the incidence of gastric cancer has declined steadily over the past two decades (Franceschi et al, 1994), predominantly by reduction in the number of tumours of the antrum (Rios-Castellanos et al, 1992), the overall 5-year survival rate remains in the range of 5–20% with little evidence of improvement despite the introduction of intensive multi-modality therapies (Kelsen et al, 1996). Surgical resection is the only potentially curative therapy, although there is continuing debate about the role of more radical surgery including extensive lymph node dissection (Cuschieri et al, 1996). Combination chemotherapy with cytotoxic agents including 5-fluorouracil (5-FU), mitomycin C, doxorubicin, methotrexate, etoposide and cisplatin, is a moderately effective means of reducing tumour bulk in advanced disease, but is not associated with any significant survival benefits in an adjuvant setting, following complete resection of gastric cancer (Wils, 1996).

The evaluation of $H_2$-receptor antagonists as anticancer agents followed from anecdotal observations of tumour regression seen in patients with ulcerating gastric cancer and mycosis fungoides (Taylor et al, 1988). The postulated mechanisms of action are mediated by $H_2$-receptor blockade of suppressor T-lymphocytes, leading to their functional inhibition and stimulation of natural killer cell activity (Griswold et al, 1984; Kikuchi et al, 1986) and antagonism of histamine-stimulated growth (Watson et al, 1993).

Tonnesen et al (1988) conducted a randomized trial and reported that cimetidine appeared to improve survival in gastric cancer patients. The British Stomach Cancer Group (BSCG) established a prospective, double-blind, placebo-controlled trial of cimetidine in all stages of gastric cancer, to refute or confirm the observation.

*Received 28 April 1999*
*Accepted 8 July 1999*

*Correspondence to:* MJS Langman

### PATIENTS AND METHODS

Between February 1990 and March 1995, 442 patients were recruited by 59 consultants from 39 hospitals. Each hospital obtained ethical approval from their local ethics committee before entering patients into the trial. Patients were eligible if they had biopsy-proven carcinoma of the stomach and were able to swallow the medication. There was no specific age limit but patients had to be fit enough to be entered into the trial and this was defined as a life expectancy greater than 3 months. Patients who had concurrent non-gastric malignancies or ill health due to other causes, which would limit their prognosis, were excluded. The previous use of $H_2$-antagonists did not exclude patients from this study. Written informed consent was gained from all cases.

Data were collected on depth of penetration, resection line involvement, the absence or presence of lymph node involvement, and distant metastases in order to allow staging according to the Birmingham system (Allum et al, 1989*a*). Randomization was by phone call to the CRC Trials Unit in Birmingham where the eligibility was checked prior to treatment allocation. Treatments were allocated to patients in a randomized, double-blind manner with the clinician, pharmacy and patients unaware of the treatment allocation. After randomization, Smith-Kline Beecham dispatched the next sealed treatment pack to the hospital pharmacy. The placebo tablets were identical in appearance and taste to cimetidine tablets; hence, only Smith-Kline Beecham knew the allocated treatment.

**Table 1** Patient characteristics on entry to the trial

| | Treatment group | | | | |
|---|---|---|---|---|---|
| | **Cimetidine 800 mg** | **Cimetidine 400 mg** | **Placebo 800 mg** | **Placebo 400 mg** | **Total** |
| Number randomized | 107 | 111 | 109 | 115 | 442 |
| Number ineligible | 1 | 2 | 2 | 2 | 7 |
| Number for analysis | 106 | 109 | 107 | 113 | 435 |
| Sex (%) | | | | | |
| Male | 77 (73) | 75 (69) | 77 (72) | 84 (74) | 313 (72) |
| Female | 29 (27) | 34 (31) | 30 (28) | 29 (26) | 122 (28) |
| Age | | | | | |
| Median | 67 | 68 | 69 | 67 | 68 |
| Inter-quartile range | 61–76 | 61–76 | 62–75 | 60–75 | 61–75 |
| Range | 33–88 | 40–85 | 36–85 | 23–86 | 23–88 |
| Stage (%) | | | | | |
| I | 3 (3) | 8 (7.5) | 3 (3) | 5 (5) | 19 (4) |
| II | 15 (14) | 17 (16) | 16 (15) | 19 (17) | 67 (16) |
| III | 31 (30) | 39 (37) | 41 (39) | 29 (26) | 140 (33) |
| IVa | 19 (18) | 8 (7.5) | 20 (19) | 21 (19) | 68 (16) |
| IVb | 36 (35) | 34 (32) | 26 (24) | 37 (33) | 133 (31) |
| Previous $H_2$-receptor antagonist (%) | | | | | |
| Yes | 36 (37) | 31 (32) | 33 (35) | 38 (39) | 138 (36) |
| No | 62 (63) | 65 (68) | 61 (65) | 59 (61) | 247 (64) |
| Other drugs (%) | | | | | |
| Yes | 17 (17) | 19 (19) | 19 (19) | 15 (14) | 70 (18) |
| No | 82 (83) | 79 (81) | 81 (81) | 88 (86) | 330 (82) |
| Time (in days) from diagnosis to randomization | | | | | |
| Median | 37 | 36 | 37 | 36 | 36 |
| Inter-quartile range | 20–56 | 24–52 | 23–56 | 22–54 | 22–55 |
| Range | 0–699 | 2–464 | 0–145 | 0–109 | 0–699 |
| Time (in days) from operation to randomization | | | | | |
| Median | 11 | 12 | 12 | 13 | 12 |
| Inter-quartile range | 7–20 | 8–28 | 9–20 | 8–24 | 8–23 |
| Range | 0–68 | 1–89 | 0–90 | 0–74 | 0–90 |
| Type of operation (%) | | | | | |
| Curative resection | 50 (47) | 58 (55) | 58 (55) | 60 (55) | 226 (53) |
| Palliative resection | 19 (18) | 25 (23) | 23 (22) | 29 (26) | 96 (22) |
| Inoperable | 37 (35) | 23 (22) | 24 (23) | 21 (19) | 105 (25) |

In general, patients were randomized a few days prior to discharge from hospital following confirmation of the diagnosis of gastric adenocarcinoma, and drugs were available as a 'take home' prescription.

Patients were randomized to receive tablets of either placebo or cimetidine in doses of 800 or 400 mg, administered twice daily until tumour progression, recurrence or death. No restriction was placed on clinical management of the patients except that concurrent known $H_2$-antagonist treatment was not allowed.

Sixty-one months after the study had been initiated, having recruited 442 patients, it proved impossible to maintain supplies of the placebo tablets. The trial data monitoring committee agreed that the blinded treatment code needed to be broken. This was achieved by matching the treatment allocation held at Smith-Kline Beecham with the randomization list and blinded treatment pack code held at the CRC Trials Unit. Patients continuing on the placebo arm were discontinued from treatment. Those on active medication with cimetidine continued as per protocol with drugs administered through pharmacy. At the time the code was broken, 71 cimetidine recipients and 67 placebo recipients were still alive, of whom 42 cimetidine and 39 placebo recipients were continuing treatment.

Patients were followed up at regular intervals, according to local policy. Treatment details were collected at 3-monthly intervals for the first 2 years and 6-monthly thereafter until the death of the patient or the censor date.

### Statistical methods

The 5-year survival for all cases of gastric cancer is around 5% (Allum et al, 1989*a*). The study aimed to recruit 500 patients in order to be able to detect differences in excess of 5% at the 5% level of significance with an 80% power. The trial stopped early when 442 patients had been randomized due to lack of placebo supplies. This resulted in a slight loss of power when detecting differences in excess of 5%, as planned, but allows differences in excess of 10% to be detected with a 95% power.

The analysis was carried out using SAS statistical software (SAS Institute, SAS Circle, Cary, NC, USA). The primary end point for analysis was death. Survival was calculated from date of

© 1999 Cancer Research Campaign 

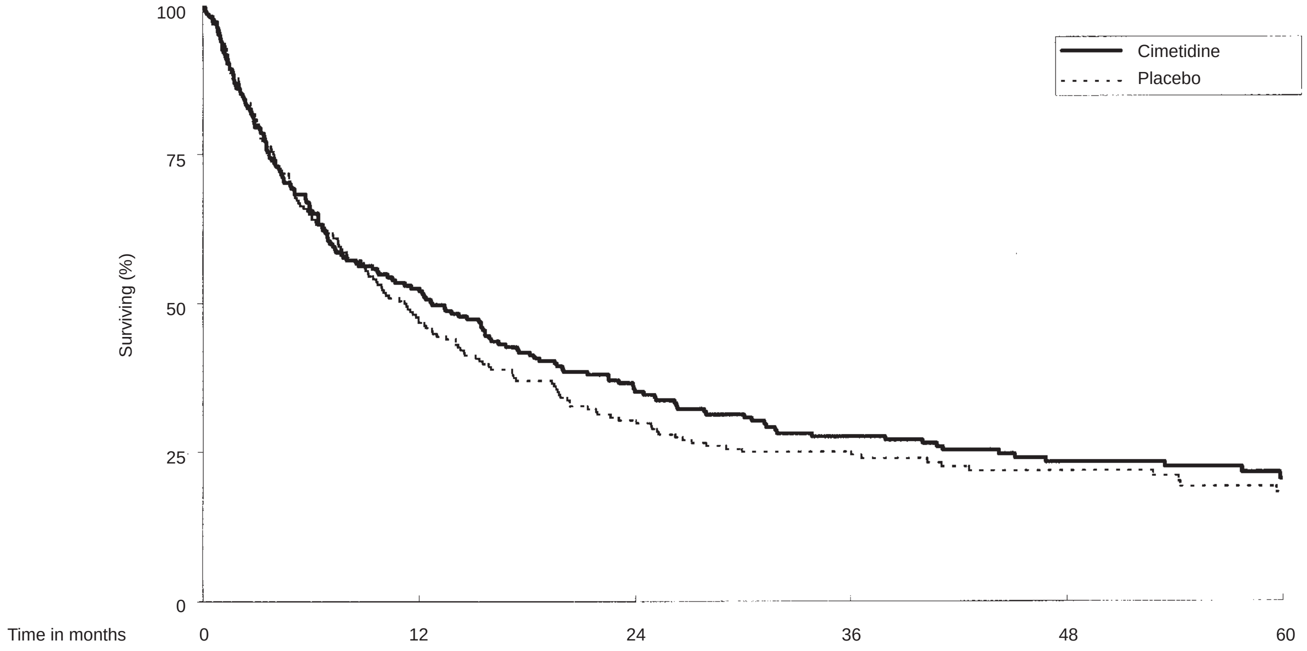

| No. at risk: | 0 | 12 | 24 | 36 | 48 | 60 |
|---|---|---|---|---|---|---|
| Cimetidine | 215 | 112 | 74 | 51 | 35 | 19 |
| Placebo | 220 | 102 | 64 | 48 | 27 | 19 |

**Figure 1** Survival analysis for each drug. Analysis of 215 patients allocated to cimetidine and 220 patients allocated to placebo ($\chi^2$ = 0.65, $P$ = 0.42)

randomization to the date of death or the censor date of 1 March 1997, when the minimum time from entry to the trial was 2 years. Survival curves were constructed using the method of Kaplan and Meier (Kaplan and Meier, 1958) and the log-rank test (Peto et al, 1977) was used to assess the differences between groups with an intention-to-treat analysis. The main comparison was between those receiving cimetidine and the placebo group. Survival was also compared for the two different doses. Survival by stage, type of resection, age and sex were also considered and treatment comparisons were stratified by these factors. Cox-proporhonal hazards models were applied to determine the independent predictors of survival.

### RESULTS

### Patients

A total of 442 patients were randomized. Seven patients were excluded from analysis because of ineligibility: two because they had oesophageal rather than gastric cancer, two with gastric lymphomas, one with concurrent bladder cancer, one did not have histological confirmation of disease and one was randomized after death. There was one protocol violator who was randomized to receive placebo but was given cimetidine. This patient was included in the analysis on an intention-to-treat basis.

All 435 eligible patients were followed up for at least 2 years, except for five patients who were lost to follow-up at 9, 10, 12, 15 and 18 months respectively. Three of these patients were known to have left the country after being diagnosed with recurrent disease. At the census date 95 patients were alive. The 75%, median and 25% times between entry to the trial and the census date were 3.0, 4.1 and 5.8 years respectively. The follow-up duration was identical in each treatment arm.

The patient characteristics are shown in Table 1. The randomization was balanced between the treatment groups in terms of sex, age and stage distribution. The median age for all eligible patients was 68 years, 72% were males. Forty-seven per cent had stage IV disease, 32% stage III, 16% stage II and 5% stage I.

### Causes of death

A total of 340 patients died during the study: 166 (49%) in the cimetidine treatment groups and 174 (51%) in the placebo groups. Three hundred and nine (91%) of the deaths were disease related: 155 in the cimetidine group and 154 in the placebo group. Two patients on cimetidine died from other cancers (pancreatic cancer and bronchial). Other causes accounted for 7% of all deaths: eight taking cimetidine and 16 having placebo. The cause of death was unknown for five patients (1% of deaths) where death dates only were obtained: one was taking cimetidine and four placebo.

### Survival

The survival curves for cimetidine and placebo recipients are shown in Figure 1. The median survival for patients receiving cimetidine was 13 months (95% confidence interval (CI) 9–16 months) and 11 months for the placebo arm (95% CI 9–14 months). There was no significant difference in survival between the two treatment groups ($\chi^2$ = 0.65, $P$ = 0.42). The 5-year actuarial survival was 21% for those randomized to cimetidine compared with 18% in the placebo arms (Table 2).

The median survival within the cimetidine group was 13 months for patients receiving 800 mg twice a day (95% CI 7–20 months) compared to 13 months (95% CI 8–18 months) for those given

 *© 1999 Cancer Research Campaign*

**Table 2** Survival analysis for treatment and prognostic factors

| | *n* | No. of deaths | % alive | O/E | Survival in months: Median | Survival in months: 95% CI | % Surviving (years): 1 | % Surviving (years): 5 |
|---|---|---|---|---|---|---|---|---|
| Treatment ($\chi^2$ = 0.65, *P* = 0.42) | | | | | | | | |
| Cimetidine | 215 | 166 | 23 | 0.96 | 13 | 9–16 | 53 | 21 |
| Placebo | 220 | 174 | 21 | 1.04 | 11 | 9–14 | 47 | 18 |
| Dose of cimetidine ($\chi^2$ = 0.51, *P* = 0.46) | | | | | | | | |
| 800 mg | 106 | 84 | 21 | 1.06 | 13 | 7–20 | 53 | 16 |
| 400 mg | 109 | 82 | 25 | 0.95 | 13 | 8–18 | 52 | 25 |
| Dose of placebo ($\chi^2$ = 0.04, *P* = 0.84) | | | | | | | | |
| 800 mg | 107 | 86 | 20 | 0.99 | 12 | 9–17 | 48 | 15 |
| 400 mg | 113 | 88 | 22 | 1.01 | 10 | 6–14 | 46 | 21 |
| Sex ($\chi^2$ = 1.29, *P* = 0.26) | | | | | | | | |
| Male | 313 | 246 | 21 | 1.04 | 11 | 8–13 | 47 | 19 |
| Female | 122 | 94 | 23 | 0.91 | 14 | 10–21 | 57 | 20 |
| Age (years) ($\chi^2$ = 7.78, *P* = 0.005) | | | | | | | | |
| Under 60 | 85 | 57 | 33 | 0.73 | 20 | 13–38 | 61 | 29 |
| 60+ | 350 | 283 | 19 | 1.08 | 11 | 8–13 | 47 | 17 |
| Stage ($\chi_{trend}^2$ = 169.36, *P* < 0.00001)[b] | | | | | | | | |
| I | 19 | 3 | 84 | 0.11 | [a] | –[a] | 95 | 78 |
| II | 67 | 35 | 48 | 0.42 | 54 | 26–[a] | 88 | 45 |
| III | 140 | 108 | 23 | 0.87 | 16 | 13–20 | 63 | 20 |
| IVa | 68 | 58 | 15 | 1.17 | 10 | 7–16 | 42 | 10 |
| IVb | 133 | 128 | 4 | 2.63 | 3 | 3–4 | 17 | 4 |
| Resection ($\chi_{trend}^2$ = 157.59, *P* < 0.00001)[b] | | | | | | | | |
| Curative resection | 226 | 146 | 35 | 0.62 | 24 | 20–31 | 73 | 32 |
| Palliative resection | 96 | 84 | 13 | 1.37 | 7 | 5–10 | 35 | 10 |
| Inoperable | 105 | 102 | 3 | 2.77 | 3 | 2–4 | 16 | 2 |
| Surgery with curative intent (stages I–III) | | | | | | | | |
| Drug ($\chi^2$ = 0.60, *P* = 0.44) | | | | | | | | |
| Cimetidine | 113 | 72 | 36 | 0.94 | 26 | 20–41 | 75 | 34 |
| Placebo | 113 | 74 | 35 | 1.05 | 20 | 16–28 | 71 | 30 |

[a]Survival estimate for confidence interval not yet reached due to too few events. [b]Log-rank test for trend.

400 mg twice a day. There was no survival difference between the two doses of cimetidine ($\chi^2$ = 0.51, *P* = 0.46). The 5-year actuarial survival was 16% for the high dose regime in comparison with 25% on the low dose (Figure 2).

Age (< 60, ≥ 60 years, *P* = 0.005), stage (I, II, III, IVa, IVb, *P* = 0.0001) and type of resection (curative, palliative, inoperable, *P* = 0.0001) were shown to be significant predictors of survival with younger, early stage patients having curative resection tending to have a better survival. However, there was no survival difference for sex (*P* = 0.26) (Table 2). Stratifying by stage, type of resection, age or sex revealed no significant differences either between treatment and placebo recipients, or between those given the higher and lower doses of cimetidine. Cox regression identified stage as the most important predictor of survival with age as the only other independent factor; stage can be substituted by type of resection. In all Cox regression analyses treatment was considered but did not enter the model.

Patients were subdivided as to whether they underwent surgery with potentially curative (stage I–III) or palliative intent or were considered inoperable. In those undergoing potentially curative resection, the median survival for the cimetidine group was 26 months (95% CI 20–41 months) compared to 20 months (95% CI 16–28 months) for the placebo group (*P* = 0.44). For those undergoing palliative resection the median survival was 7 months for both the cimetidine and placebo groups (95% CI 4–18 months and 5–10 months respectively). Similarly, for the inoperable patients median survival was 3 months (95% CI 2–5 months) for both the cimetidine and placebo group. Stratifying by type of resection (Figure 3) showed no significant survival advantage for cimetidine (*P* = 0.48).

### DISCUSSION

Over the past 50 years there has been a significant decline in the incidence of gastric cancer in males and females in Western societies. The disease is rare before the age of 40 years but its incidence rises steadily to reach a peak in the seventh decade. Although the precise aetiology is unknown, environmental and genetic factors have been identified which contribute to its development (Fuchs and Mayer, 1995). Attempts to improve survival rates have focused on earlier diagnosis, radical surgery and adjuvant chemo-radiotherapy.

The BSCG has conducted two trials of adjuvant therapy in the past. In the first it was not possible to show a benefit from 2 years of dual-agent chemotherapy (mitomycin C + 5-FU) with or without induction chemotherapy (Allum et al, 1989*b*). The second

*© 1999 Cancer Research Campaign* 

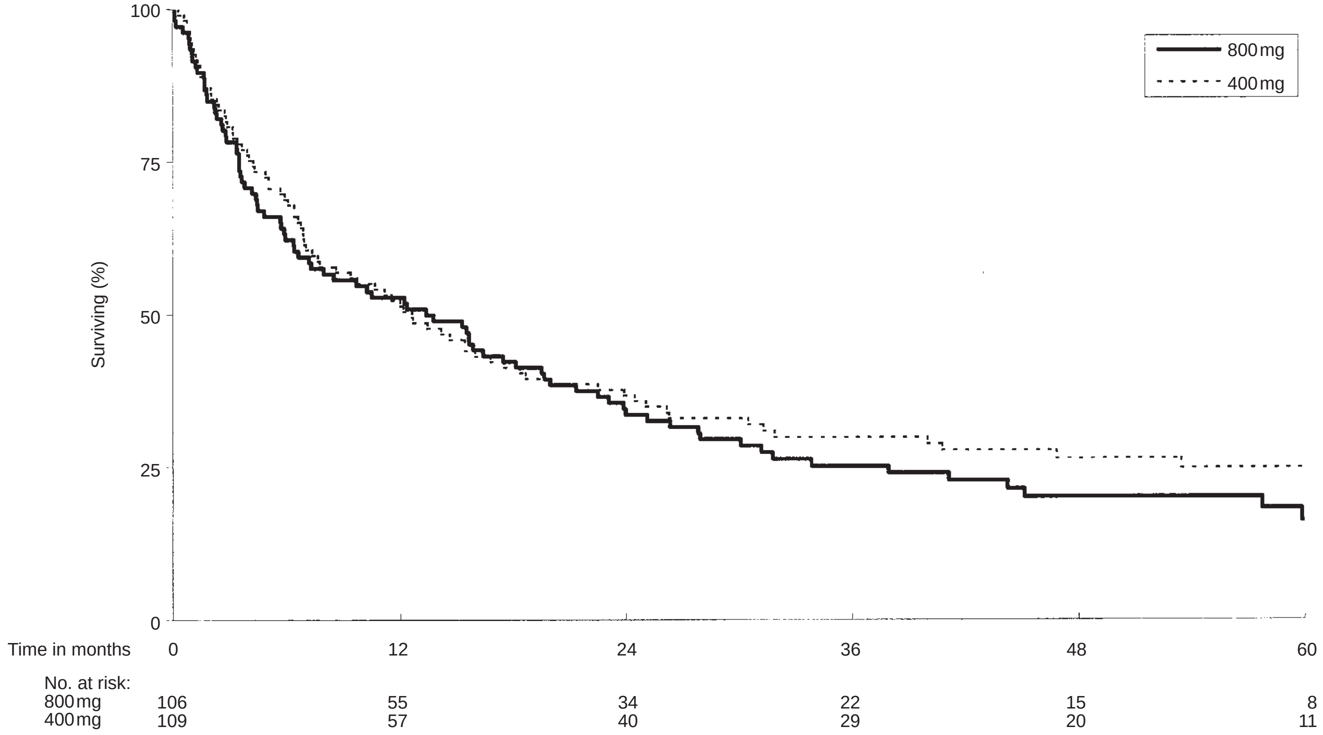

**Figure 2** Survival analysis for cimetidine by dose. Analysis of 106 patients allocated to 800 mg and 109 patients allocated to 400 mg cimetidine ($\chi^2$ = 0.51, *P* = 0.46)

trial showed no benefit from a 6-month course of triple-agent chemotherapy (mitomycin C, adriamycin and 5-FU) or radiotherapy (4500 Gy) over surgery alone (Hallissey et al, 1994). These trials reflected both the lack of survival benefit and the significant haematological and renal toxicity seen in several other trials (Wils, 1996). A meta-analysis of a number of studies has also failed to demonstrate survival benefits with conventional cytotoxic agents (Hermans et al, 1993).

The $H_2$-receptor antagonist cimetidine is well tolerated, anecdotally linked to healing of malignant gastric ulcers and has been shown to inhibit suppressor T-lymphocyte function (Griswold et al, 1984; Kikuchi et al, 1986), leading to suggestions that it could have a useful role as an anticancer agent. We have failed to replicate the findings of Tonneson et al (1988), who reported median survival to be improved from 316 days in placebo recipients to 450 days in those given cimetidine 400 mg twice daily, with a corresponding improvement in relative survival rates from 28% to 45% at 1 year. In a study of 222 gastric cancer patients, median survival appeared markedly greater in those receiving ranitidine 150 mg twice daily (331 days, 95% CI 232–393 days) compared to those receiving placebo treatment (187 days, 95% CI 143–269 days) (Primose et al, 1998). However, the median follow-up was only 6 months and the result was non-significant (*P* = 0.23). Overall 73 of 107 ranitidine recipients with staged tumours died compared with 77 of 109 placebo recipients, the small difference being at least accountable for by a higher proportion of stage I and II cases allocated ranitidine. Our study is larger, was able to detect a 10% improvement with 95% power, and the period of follow-up was considerably more prolonged with a median of 4 years. Any difference in survival between cimetidine and placebo recipients in our study is accounted for by a greater (but non-significant) number of non-cancer deaths in placebo recipients, and the actual number of cancer deaths are identical in cimetidine and placebo recipients. Stratifying for stage or looking at the curative (stages I–III) and palliative (stages IVa–IVb) patients, also failed to find survival benefits for cimetidine. Finally, our study showed no evidence of a dose related difference in survival, as might have been expected if cimetidine were effective.

Given cimetidine's inhibition of suppressor T-cells, stimulation of cell-mediated immunity and the demonstration that histamine receptors are expressed on a range of cancer cell lines which mediate cimetidine's direct anti-proliferative effects in vitro, it is hardly surprising that there are other clinical studies examining in different tumour types its anticancer properties. Burtin et al (1988) performed a small randomized study of 65 patients with a range of advanced refractory tumours, who were assigned best supportive care or combination therapy with cimetidine and subcutaneous injections of histamine. The authors reported improved survival with the combination regime (172 vs 26 days) and there was a suggestion that quality of life was also improved. Cimetidine has also been incorporated into a complex chemo-immunotherapeutic regime (Gold et al, 1993) which combined infusion of auto-lymphocytes (ex vivo activated peripheral blood lymphocytes) from tumour bearing hosts, with cimetidine and cyclophosphamide. An early report of activity associated with this regime (25% response rate in 20 patients with refractory solid tumours) has not encouraged other groups to take this therapy forward into randomized trials.

 *© 1999 Cancer Research Campaign*

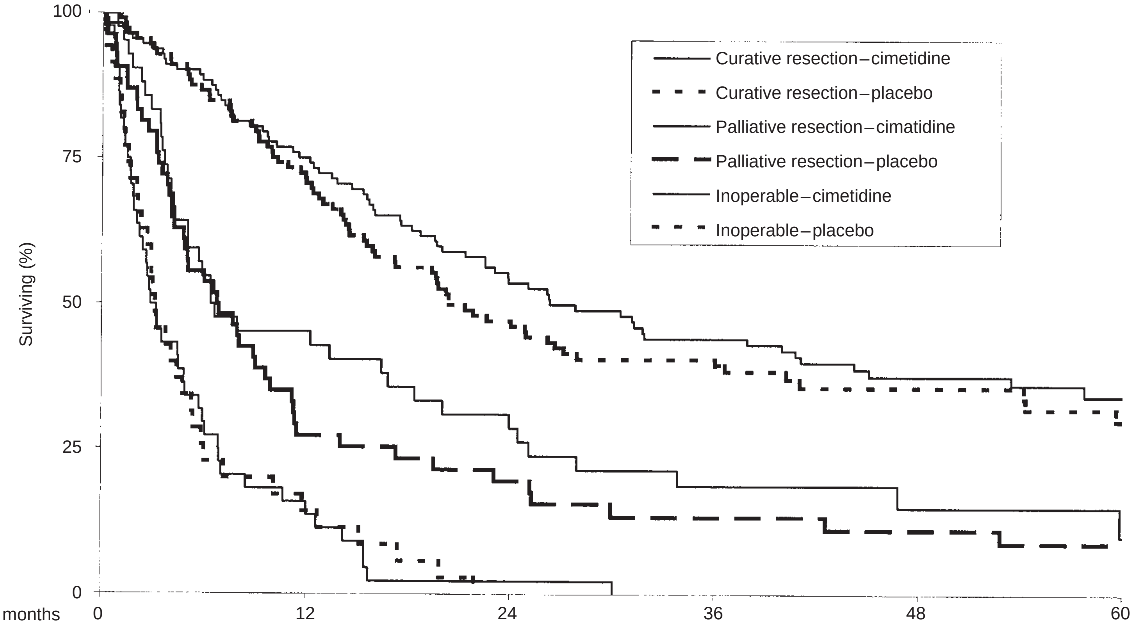

| No. at risk: | 0 | 12 | 24 | 36 | 48 | 60 |
|---|---|---|---|---|---|---|
| Curative resection–cimetidine | 113 | 84 | 59 | 44 | 31 | 17 |
| Curative resection–placebo | 113 | 80 | 51 | 41 | 21 | 15 |
| Palliative resection–cimetidine | 42 | 19 | 12 | 7 | 4 | 2 |
| Palliative resection–placebo | 54 | 14 | 10 | 6 | 5 | 3 |
| Inoperable–cimetidine | 55 | 9 | 3 | 0 | 0 | 0 |
| Inoperable–placebo | 50 | 8 | 0 | 0 | 0 | 0 |

**Figure 3** Survival by drug stratifying by type of resection ($\chi^2 = 0.51$, $P = 0.48$)

Renal cell cancer is somewhat responsive to treatment with the cytokine, α-interferon and is considered an appropriate therapeutic target for novel immunomodulatory agents. On the basis of two earlier phase II trials in renal cancer, suggesting that cimetidine induced a tumour response in 20–30% of patients, Sagaster et al (1995) conducted a randomized trial in 148 patients with metastatic renal cancer. Patients received either interferon α (5 MU 5 × weekly, subcutaneously) or interferon plus coumarin (100 mg daily) and cimetidine (1200 mg daily). No differences were found in remission rates or survival times suggesting that coumarin and cimetidine do not enhance the anti-tumour properties of interferon.

There are interesting data in colorectal cancer, with a small trial of 35 patients with advanced disease randomized to receive 5-FU-based chemotherapy alone or in combination with cimetidine (400 mg twice daily) (Links et al, 1995). There was no difference in overall response; however, the authors demonstrated that there was a higher incidence of reduction (> 50% baseline) of the tumour marker, carcinoembryonic antigen, in the cimetidine-treated group. The same investigators also performed a small randomized trial in which 42 patients scheduled for elective removal of colorectal cancers were randomized to receive cimetidine (400 mg twice daily for 1 week) or control (Adams et al, 1997). They examined lymphocyte infiltration in the resected tumour specimen and found a significantly higher lymphocyte response in the cimetidine treated group (56% vs 21%). They also showed that there is a positive relationship between lymphocyte infiltration and survival, providing some surrogate of evidence of the efficacy of cimetidine. A larger randomized trial of 192 colorectal cancer patients undergoing palliative or curative resections was unable to demonstrate any survival benefit for the cimetidine group (Svendsen et al, 1995).

In summary, on the basis of the present study, we conclude that there is still not enough evidence to show that $H_2$-antagonist treatment is an effective therapy for patients with gastric cancer.

### ACKNOWLEDGEMENTS

The authors acknowledge the financial support of the Cancer Research Campaign and Smith-Kline Beecham. Trial co-ordination and data management were carried out by Mrs Patricia Baker and Mrs Jackie Davies, CRC Trials Unit, Birmingham. We thank members of the British Stomach Cancer Group for contributing patients and the West Midlands Cancer Intelligence Unit for their help.

### REFERENCES

Adams WJ and Morris DL (1997) Pilot study–cimetidine enhances lymphocyte infiltration of human colorectal carcinoma: results of a small randomized control trial. *Cancer* **80**: 15–21

Allum WH, Powell DJ, McConkey CC and Fielding JW (1989a) Gastric cancer: a 25-year review. *Br J Surg* **76**: 535–540

*© 1999 Cancer Research Campaign*

Allum WH, Hallissey MT and Kelly KA (1989b) Adjuvant chemotherapy in operable gastric cancer. 5 year follow-up of first British Stomach Cancer Group trial. *Lancet* **1**: 571–574

Burtin C, Noirot C, Scheinmann, Galoppin L, Sabolovic D and Bernard P (1988) Clinical improvement in advanced cancer disease after treatment combining histamine and H2 antihistaminics (ranitidine or cimetidine). *Eur J Cancer Clin Oncol* **24**: 161–167

Cuschieri A, Fayers P, Fielding J, et al. (1996) Postoperative morbidity and mortality after D1 and D2 resections for gastric cancer: preliminary results of the MRC randomised controlled surgical trial. The Surgical Cooperative Group. *Lancet* **347**: 995–999.

Franceschi S, Levi F and La Vecchia C (1994) Epidemiology of gastric cancer in Europe. *Eur J Cancer Prev* **3**: 5–10.

Fuchs CS and Mayer RJ (1995) Gastric carcinoma. *N Engl J Med* **333**: 32–41.

Gold GE, Malamud SC, LaRosa F, Seder R and Osband ME (1993) Adoptive chemoimmunotherapy for the treatment of relapsed and refractory solid tumors using ex vivo activated memory T cells (autolymphocyte therapy) and cyclophosphamide. *J Immunother* **13**: 213–221.

Griswold DE, Alessi S, Badger AM, Poste G and Hanna N (1984) Inhibition of T suppressor cell expression by histamine type 2 (H2) receptor antagonists. *J Immunol* **132**: 3054–3057

Hallissey MT, Dunn JA, Ward LC and Allum WH (1994) The second British Stomach Cancer Group trial of adjuvant radiotherapy or chemotherapy in resectable gastric cancer: five-year follow-up. *Lancet* **343**: 1309–1312.

Hermans J, Bonenkamp JJ, Boon MC, Bunt AMG, Ohyama S, Sasako M and Van de Velde CHJ (1993) Adjuvant therapy after curative resection for gastric cancer: meta-analysis of randomised trials. *J Clin Oncol* **11**: 1441–1447.

Kaplan EL and Meier P (1958) Non parametric estimation from incomplete observations. *J Am Stat Assoc* **53**: 457

Kelsen DP (1996) Adjuvant and neoadjuvant therapy for gastric cancer. *Semin Oncol* **23**: 379–389.

Kikuchi Y, Oomori K, Kizawa I and Kato K (1986) Augmented natural killer activity in ovarian cancer patients treated with cimetidine. *Eur J Cancer Clin Oncol* **22**: 1037–1043

Links M, Clingan PR, Phadke K, O'Baugh J, Legge J, Adams WJ, Ross WB and Morris DL (1995) A randomised trial of cimetidine with 5-fluorouracil and folinic acid in metastatic colorectal cancer. *Eur J Surg Oncol* **21**: 523–525.

Peto R, Pike MC, Armitage P, Breslow NE, Cox DR, Howard SV, Mantel N, McPherson K, Peto J and Smith PG (1977) Design and analysis of randomized clinical trials requiring prolonged observation of each patient. II. Analysis and example. *Br J Cancer* **35**: 1–39.

Primrose JN, Miller GV, Preston SR, Gokhale J, Ambrose NS, Ward UM, Mills JG, Ehsanullah RSB, Darekar B and the Yorkshire GI Tumour Group (1998) A prospective randomised controlled study of the use of ranitidine in patients with gastric cancer. *Gut* **42**: 17–19.

Rios-Castellanos E, Sitas F, Shepherd NA and Jewell DP (1992) Changing pattern of gastric cancer in Oxfordshire. *Gut* **33**: 1312–1317.

Sagaster P, Micksche M, Flamm J and Ludwig H (1995) Randomised study using IFN-alpha versus IFN-alpha plus coumarin and cimetidine for treatment of advanced renal cell cancer. *Ann Oncol* **6**: 999–1003

Svendsen LB, Ross C, Knigge U, Frederiksen HJ, Graversen P, Kjaergard J, Luke M, Stimpel H and Sparso BH (1995) Cimetidine as an adjuvant treatment in colorectal cancer. A double-blind, randomized pilot study. *Dis Colon Rectum* **38**: 514–518.

Taylor TV, Boom SJ, Blower AL, McMahon RF and Lawler W (1988) Healing of a malignant gastric ulcer with cimetidine. *J R Coll Surg Edin* **33**: 339–340.

Tonnesen H, Knigge U, Bulow S, et al. (1988) Effect of cimetidine on survival after gastric cancer. *Lancet* **2**: 990–992.

Watson SA, Wilkinson LJ, Robertson JR and Hardcastle JD (1993) Effect of histamine on the growth of human gastro-intestinal tumours: reversal by cimetidine. *Gut* **34**: 1091–1096

Wils J (1996) The treatment of advanced gastric cancer. *Semin Oncol* **23**: 397–406

*© 1999 Cancer Research Campaign*
